# Non-Invasive Assessment of Endothelial Shear Stress in Myocardial Bridges Using Coronary Computed Tomography Angiography

**DOI:** 10.1177/00033197231156637

**Published:** 2023-02-14

**Authors:** Andreas A. Giannopoulos, Basil Bolt, Dominik C. Benz, Michael Messerli, Elia Von Felten, Dimitri Patriki, Catherine Gebhard, Aju P. Pazhenkottil, Christoph Gräni, Philipp A. Kaufmann, Ronny R. Buechel, Oliver Gaemperli

**Affiliations:** 1 Department of Nuclear Medicine, Cardiac Imaging, 31001 University Hospital Zurich, Zurich, Switzerland; 2 Department of Cardiology, 30362 Inselspital Bern, Bern, Switzerland; 3 60319 Hirslanden Heart Clinic, Zürich, Switzerland

**Keywords:** coronary atherosclerosis, endothelial shear stress, coronary computed tomography angiography, computational fluid dynamics, myocardial bridging

## Abstract

Myocardial bridging (MB) is a segment of coronary arteries with an intramural course, typically spared from atherosclerosis, while the adjacent proximal segment is reported to be atherosclerosis-prone, a phenomenon contributed to local endothelial shear stress (ESS). We aimed to describe the ESS milieu in coronaries with MBs combining coronary computed tomography angiography with computational fluid dynamics and to investigate the association of atherosclerosis presence proximal to MBs with hemorheological characteristics. Patients (*n* = 36) were identified and 36 arteries with MBs (11 deep and 25 superficial) were analyzed. ESS did not fluctuate 5 mm proximally to MBs vs 5 mm within MBs (0.94 vs 1.06 Pa, *p* = .56). There was no difference when comparing ESS in the proximal versus mid versus distal MB segments (1.48 vs 1.37 vs 1.9 Pa, p = ns). In arteries with plaques (*n* = 12), no significant ESS variances were observed around the MB entrance, when analyzing all arteries (*p* = .81) and irrespective of morphological features of the bridged segment (deep MBs; *p* = .65, superficial MBs; *p* = .84). MBs are characterized by homogeneous, atheroprotective ESS, possibly explaining the absence of atherosclerosis within bridged segments. The interplay between ESS and atherosclerosis is potentially not different in arteries with MB compared with arteries without bridges.

## Introduction

Myocardial bridging (MB), defined as a segment of a coronary artery that runs intramurally through the myocardium, is considered a usually benign coronary anomaly that is not associated with worse outcomes in patients without obstructive coronary artery disease (CAD).^1^ Most commonly an incidental finding in pathology studies and diagnostic angiographies (invasive or non-invasive), appreciation of MBs has increased in the last decades primarily due to the intensified use of coronary computed tomography angiography (CCTA) that is considered the reference standard for its non-invasive detection.^2,3^

The left coronary system is primarily involved and mainly the left anterior descending coronary artery (LAD), while the right coronary artery is rarely affected.^4^ The proximal arterial segment entering the MB is reported to frequently show atherosclerotic plaque formation, while interestingly the MB segment itself is typically spared from atherosclerosis.^5–7^ Several mechanisms have been proposed, among which complex biomechanical factors affecting the segment proximally and at the entrance of the bridge. These include myocardial systolic compression of MBs and the resulting alteration of local blood flow, as well as alteration in coronary vasoreactivity, mechanical forces (e.g., tensile stress), and the lack of perivascular adipose tissue that might be atheroprotective.^5,6,8,9^

Endothelial shear stress (ESS), a local hemodynamic factor that plays a key role in the natural history of atherosclerosis, has been also postulated to contribute to the pathophysiological changes in arteries with bridged segments.^5,10^ Coronary segments that are exposed to low ESS have been shown to exert a predisposition to atherosclerosis by inducing a proinflammatory and proatherogenic local environment.^11^ In contrast, high ESS is considered to be implicated at a later stage of coronary atherosclerosis and its presence is associated with the susceptibility of plaques to rupture or erode.^12,13^ The predilection for atherosclerotic changes of the coronary segments proximal to MBs and the lack of disease within the bridged segment has been attributed also to ESS, mainly in small studies and theoretical work.^8,14,15^

The present study aimed to describe the global and local ESS patterns in arteries with MBs and to investigate the association of atherosclerosis presence at the segment proximal to a MB with hemorheological characteristics of coronary arteries.

## Methods

Consecutive patients who underwent a clinically indicated CCTA at the University Hospital Zurich between March 2016 and May 2017 and with the presence of MB as mentioned in the clinical CCTA report were retrospectively identified. The study protocol was approved by the local ethics committee (BASEC-Nr. 2018-00508), and only subjects that signed the general informed consent were included. Written informed consent refusing use of data was considered an exclusion criterion.

### CCTA and Assessment of Anatomical Features

Contrast-enhanced CCTA was performed on a 256-slice CT scanner (CT Revolution, GE Healthcare, Chicago, Illinois, USA), using axial scanning with prospective ECG triggering, as previously described.^16,17^ Patients with a heart rate >65 beats/min received oral intravenous metoprolol premedication (Beloc, AstraZeneca, London, UK) intravenously. All patients received 0.4 mg of sublingual isosorbide dinitrate (Isoket, Schwarz Pharma, Monheim, Germany). The following acquisition parameters were used: collimation 256 × 0.625 mm with an axial resolution of 0.23 × 0.23 mm, z-coverage 160 mm, and gantry rotation time 280 ms. All scans were acquired at the mid-diastolic phase of the RR cycle.

CCTA images were evaluated with dedicated software (CardIQ Xpress-Auto Coronary Analysis, GE Healthcare, Chicago, Illinois, USA) by a board-certified cardiologist with European Association of Cardiovascular Imaging (EACVI) Level 3 cardiac CT certification and 8 years of experience in cardiac imaging (AAG). Multiplanar reconstruction images and cross-sectional views of the coronary arteries were used to verify the presence and assess the anatomical features of MB, as well as to identify plaques proximal to the MBs. To describe the morphological characteristics of bridged segments, the distance (in mm) from the ostium to the entrance of the MB and the length (in mm) of the coronary segment beneath the MB (from the entrance to exit) were measured. MBs were categorized based on the depth of the tunneled segment beneath the epicardial surface, as superficial when ≤2 mm and deep when >2 mm.^7,18,19^ Plaques were characterized visually and based on Hounsfield unit (HU) categories: 100 to 130 HU (low attenuation; non-calcified), 131 to 350 HU (mixed), and >350 HU (calcified). The degree of luminal stenosis was assessed visually and lesions were divided into stenotic (>50%) and non-stenotic (<50%), based on area percentage stenosis.

### ESS Calculation

The coronary lumen was semi-automatically segmented with manual editing when necessary and reconstructed in three-dimensional (3D) space (Mimics, Materialise NV, Leuven, Belgium) resulting in standard tessellation language (STL) models of the endoluminal surface of the coronary arteries. ESS was calculated using a computational fluid dynamics approach (Ansys Inc, Canonsburg, PA, USA) with patient-specific boundary conditions as previously described.^20^ Tetrahedral computational meshes of the volumes enclosed by each STL model were created in ICEM (version 15.0, Ansys Inc, Canonsburg, PA, USA). The meshes were generated using a 0.2 mm internal element size and 3 prismatic layers with an initial layer height of 20 μm and exponential growth factor of 1.2 to capture near wall effects, and conversion of tetrahedral in polyhedral mesh was subsequently performed. The incompressible Navier–Stokes equations were solved for steady-state flow and considering blood a Newtonian fluid with a constant density of 1058 kg/m^3^. The total inlet flow rate was calculated based, for each patient, on myocardial mass assuming myocardium rest perfusion at a rate of 0.8 mL/min/g and was applied as a constant inlet velocity.^20,21^ Murray’s law was used to determine the relative flow distribution at coronary bifurcations.^22^

**Figure 1.**
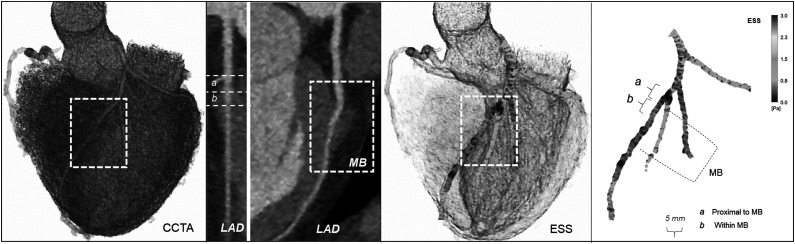
Endothelial shear stress (ESS)–profiling methodology. Coronary computed tomography angiography multiplanar reconstruction images and cross-sectional views of the coronary arteries were created and interpreted. The coronary artery lumen was segmented and reconstructed in three-dimensional space and ESS was calculated with the use of computational fluid dynamics. Local ESS values were extracted for the coronary segments of interest.

In accordance with prior pathology studies, each artery was divided into 5-mm-long segments whereby ESS was averaged and categorized as low if <1 Pascal (Pa), normal/intermediate if >1<3 Pa, and as high if >3 Pa.^7,23^ Absolute ESS values were compared for the following coronary segments: (i) from the coronary ostium to the MB, (ii) within the MB, (iii) distally to the MB, (iv) 5 mm proximal to the MB entrance, and (v) the first 5 mm within the MB. Each MB was further divided into proximal, mid, and distal segments based on length tertiles, and the respective absolute ESS values were compared. For the association with plaque presence, global [defined as coronary segments (i)–(iii)] and local [defined as coronary segments (iv)–(v)] ESS values were compared in patients with and without atherosclerosis at the segment proximal to the MB (Figure 1).

## Statistical Analysis

Statistical analyses were performed with IBM SPSS Statistics Version 21.0 (IBM Corp., New York, NY, USA) and GraphPad Prism Version 6.0 (GraphPad, Inc., San Diego, CA, USA). All variables were assessed for normality, and results were expressed as mean ± standard deviation (SD) for continuous variables and as absolute counts and percentages for categorical variables. Comparison of continuous variables was performed with t-test/Mann–Whitney-U test and using analysis of variance (ANOVA) and repeated measure ANOVA for multigroup analysis. The association of plaque presence with ESS was assessed using t-test/Mann–Whitney-U test and with χ^2^ analysis/Fisher’s exact test. A two-sided *p* < .05 was considered significant.

## Results

### Demographics and Anatomical Characteristics

Demographic parameters of the patients included in the study are presented in Table 1, and the CCTA indication for all study participants was exclusion of CAD due to atypical chest pain. Forty-eight patients with MB in the LAD were identified; 12 had to be excluded from further analysis accounting for segmentation issues due to poor image quality/artifacts. No patients were excluded due to inability to segment the coronary lumen of the bridged segments. A total of 36 arteries from 36 patients were analyzed. Morphological characteristics of the MB are described in Table 2. No bifurcations were noted arising from the bridged segments. In 12 arteries, plaques were identified proximally to the MB with the majority of the plaques being calcified (75%) while in 9 of them, the lesion was non-stenotic. No plaques were detected within the tunneled segments. Eleven of the MBs were deep with a mean depth of 2.7 mm, and in four of them, plaques were identified proximally to the bridged segment. Comparison of all morphological parameters between superficial and deep MBs yielded non-significant differences (Table 3). There was no correlation between plaque presence and MBs category (deep vs superficial, χ^2^ *p* = .80) or with the length of the MBs (*p* = .45). In arteries with plaques in proximal LAD, the arterial length proximally to the bridged segment was significantly longer compared to arteries without plaques (65.9 vs 54.2 mm; *p* = .02).

**Table 1.** Clinical characteristics of the study population (*n* = 36).

Women	10 (28%)
Age, years	56.6 ± 10.3
Risk factors	
Hyperlipidemia	22 (61.1%)
Diabetes mellitus	3 (8.3%)
Family history	12 (33.3%)
Smoking	9 (25%)
Hypertension	12 (33.3%)

Values shown are mean ± standard deviation, absolute numbers, and percentages.

**Table 2.** Characteristics of arteries with myocardial bridging (MB).

Artery pertaining MB	
Left anterior descending (LAD)	36 (100%)
MB characteristics	
Deep	11 (30.6%)
Length, mm	28.5 (14.8)
Depth, mm	1.76 (0.8)
Distance from ostium, mm	83.7 (14.9)
Plaque features	
Plaque presence	12 (33.3%)
Plaque location	
Proximal to MB	12 (100%)
Distance plaque to ostium, mm	30.6 (12.4)
Distance plaque to MB, mm	22.4 (18.7)
Calcified plaque	9 (75%)
Mixed plaque	3 (25%)
Degree of stenosis	
<50%	9 (75%)
>50%	3 (25%)

Values shown are mean ± standard deviation, absolute numbers, and percentages.

**Table 3.** Morphological characteristics of deep and superficial myocardial bridges (MBs) and of atherosclerotic plaques in the studied coronary arteries.

	Deep	Superficial	p
MB characteristics			
N	11	25	
Length, mm	27.1 (12.1)	25.4 (11.0)	.69
Depth, mm	2.6 (0.8)	1.4 (0.3)	<.001
Distance from ostium, mm	51.3 (11.2)	61.1 (15.5)	.08
Plaque features			
Plaque presence	4 (36%)	8 (32%)	
Distance plaque to ostium	36.6 (13.8)	27.5 (11.4)	.263
Distance plaque to MB	14.9 (2.4)	26.1 (22.4)	.999
Calcified plaque	3 (75%)	6 (75%)	
Mixed plaque	1 (25%)	2 (25%)	
Degree of stenosis			
<50%	3 (75%)	6 (75%)	
>50%	1 (25%)	2 (25%)	

Values given are mean ± standard deviation, absolute numbers, and percentages.

### Hemorheological Parameters

**Figure 2.**
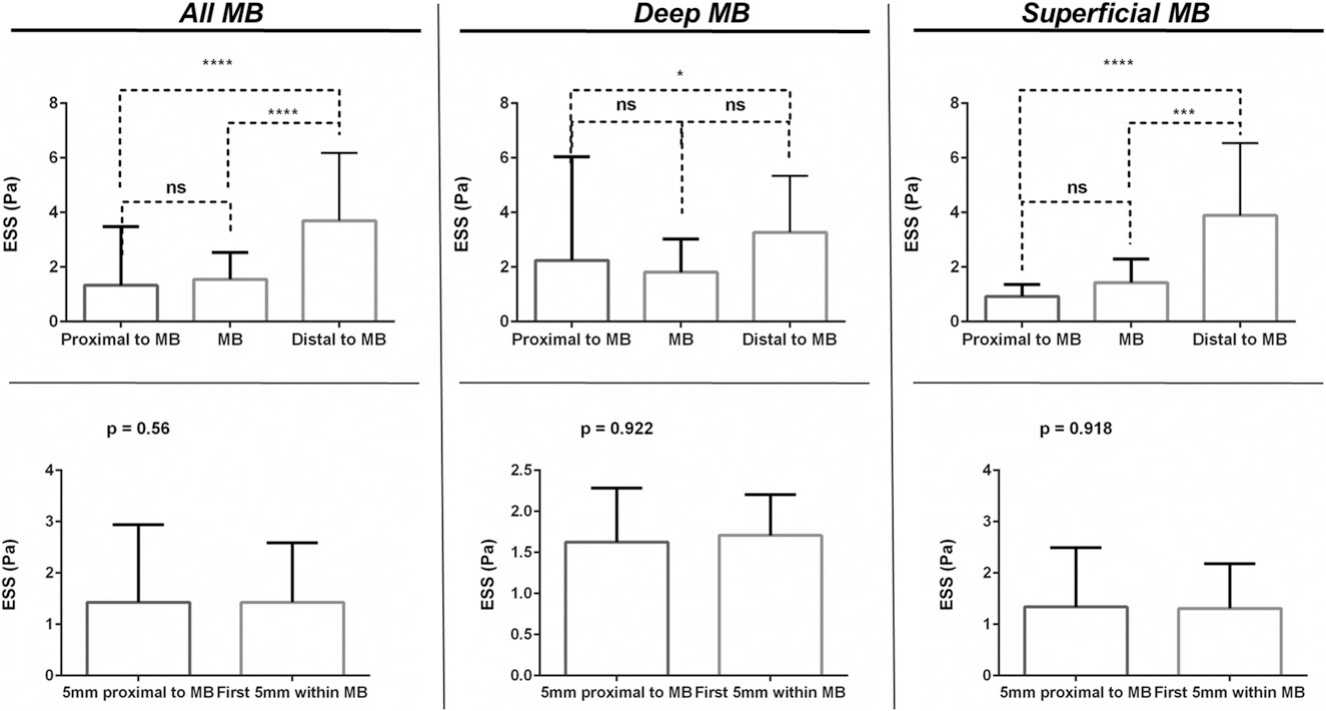
Endothelial shear stress (ESS) absolute values comparison between arterial segments in arteries with myocardial bridging (MB). Upper panels depict global endothelial shear stress (ESS) comparison between proximal to MB, within the MB and distal to MB in all arteries as well as separately in deep and superficial MBs. Lower panels show ESS categories comparison around the entrance of MBs in all arteries as well as separately in deep and superficial MBs.

Absolute ESS values differed between the MB segment and the segment distal to the MB (0.95 vs 2.98 Pa, *p* < .0001), but not between the MB segment and the segment proximal to the MB (0.95 vs 1.2 Pa, *p* = .17), an effect that was similarly observed in deep and in superficial MBs (Figure 2). ESS values 5 mm proximal to the MB were not different when compared with the first 5 mm within the MB (0.94 vs 1.06 Pa, *p* = .56) when all arteries were taken together. For deep (1.63 vs 1.71 Pa, *p* = .92) and superficial (1.34 vs 1.31 Pa, *p* = .91) MBs, no statistically significant variations of ESS were documented (Figure 2).

**Figure 3.**
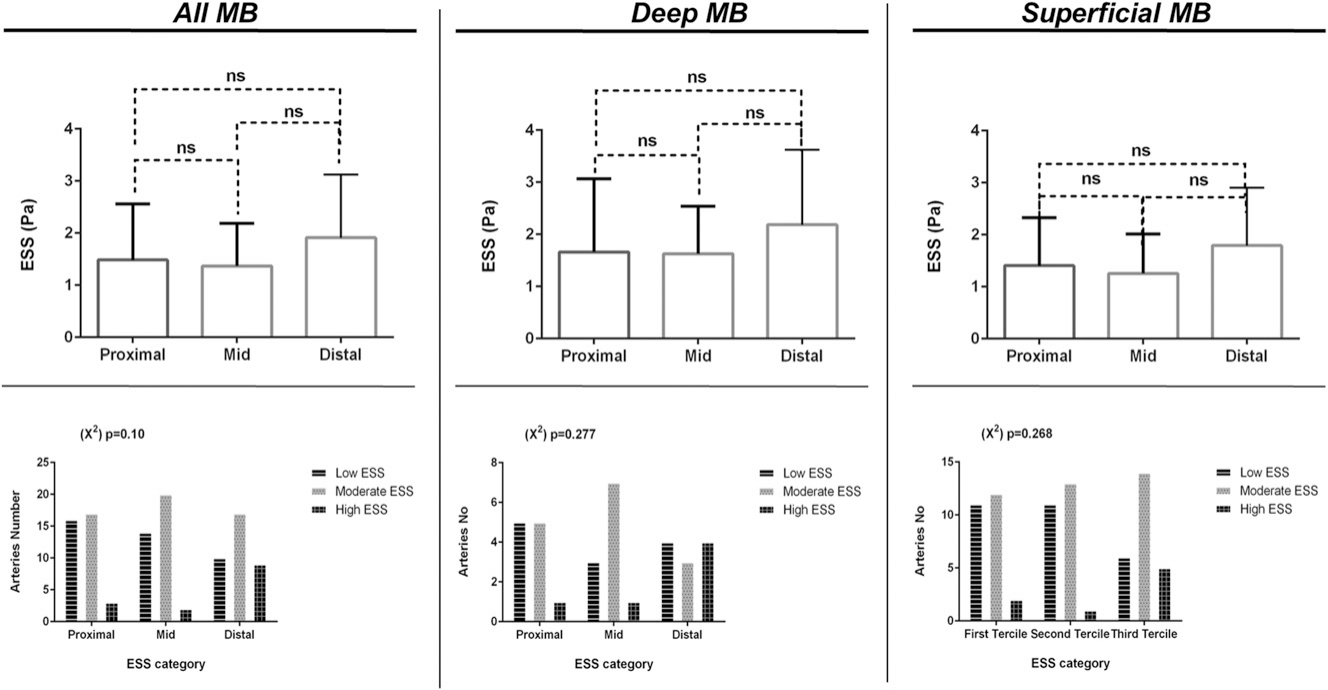
Endothelial shear stress (ESS) absolute values comparison across the length myocardial bridges (MBs). Upper panels depict absolute endothelial shear stress (ESS) values comparison between the proximal, the mid, and the distal segment of MBs in all arteries as well as separately in deep MBs and in superficial MBs. Lower panels show ESS categories comparison between the proximal, the mid, and the distal segment of MBs in all arteries as well as separately in deep and superficial MBs.

Bridged segments were characterized by homogeneous absolute ESS values throughout their length when comparing the proximal versus the mid versus the distal segments (1.48 vs 1.37 vs 1.9 Pa, all p = ns). This pattern was also demonstrated in deep (1.66 vs 1.63 vs 1.94 Pa, all p = ns) as well as in superficial (1.41 vs 1.26 vs 1.79 Pa, all p = ns) MBs. For all analyses, ESS values within the tunneled segment were predominantly within the intermediate and low range, while less frequently values of >3 Pa were observed (Figure 3).

**Figure 4.**
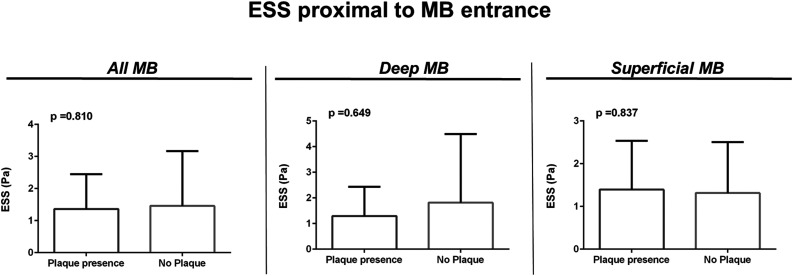
Endothelial shear stress (ESS) and plaque presence analysis. ESS absolute values comparison in the 5 mm segment proximal to the entrance of myocardial bridges (MBs). Panel on the left depicts comparison in all arteries between arteries with plaque presence versus arteries with no plaque presence. Middle panel depicts comparison in deep MBs. The right panel shows comparison in superficial MBs.

Average ESS in the segment proximally to the MB and in the bridged segment did not correlate with the presence of plaques (*p* = .23) while the absence of correlation was also noted for the ESS values 5 mm proximally to the MB compared with ESS within the first 5 mm of the MB (*p* = .39). When comparing arteries with plaque presence and without plaque presence proximally to the MB, ESS was shown to be not different proximally to the MB entrance (*p* = .81) when all arteries were analyzed together, as well as in deep MBs (*p* = .65) and in superficial MBs (*p* = .84) (Figure 4). Analysis of plaque features and degree of luminal stenosis with ESS was not statistically feasible due to the small sample size.

## Discussion

In this descriptive study, we assessed using real-world data the global and local ESS milieu of coronaries with bridged segments, based on non-invasive imaging with CCTA and patient-specific flow simulations. ESS around the entrance of MBs was not within abnormal, pathological values, and extreme variations were not observed. ESS was shown to be homogeneous and in the normal range within the bridged segments. No correlation between plaque presence proximally to the MB and ESS proximally to the bridged segment was observed. The aforementioned results were demonstrated in both superficial and deep MBs.

ESS increases distally to MBs while residing in comparable values when comparing the proximal to the MB and the MB coronary segments, a physiological phenomenon, owing to the tapering of the coronary arteries. Around the inlet of MBs, ESS was shown to be in a comparable range for both deep and superficial bridged segments, and there was no relevant variation in the local ESS. Endothelial cells immediately proximally to MBs have been shown to be dysfunctional and with altered morphology when compared with the endothelium within bridged segments, a process attributed to pathological alterations of ESS.^14^ It could be postulated that local shear stress is probably a less important factor while tensile forces due to systolic compression and perivascular differences between non-bridged and bridged segments (i.e., lack of perivascular fat) are the key mediators for this cellular phenotype.

Previous reports have suggested that low shear stress (<1 Pa) may contribute to atherosclerotic plaque formation proximal to the MB, whereas high shear stress (>3 Pa) may have a protective role within the tunneled segment.^5,8,15^ We showed here that MBs are characterized by ESS homogeneity throughout their length when comparing the proximal versus the mid versus the distal segments. This homogeneous ESS lies within the normal, atheroprotective range of 1–2 Pa, potentially attributing to the lack of atherosclerosis within the bridged segment. Physiologically, the distal MB segments have slightly higher absolute ESS values, most prominently in deep MS, which nevertheless remains within the physiological range.

No difference in the local ESS patterns around the entrance of MBs in arteries with plaques or without plaques proximal to MBs was demonstrated. Atherosclerosis has been suggested to be mainly located proximal to MBs, yet recent large studies have demonstrated that the disease burden is comparable to arteries without bridged segments, while others have reported a negative association of MBs in LAD and global atherosclerotic burden.^24–26^ These observations could potentially be explained by our findings suggesting that local and global ESS in MBs might not play a role in atherosclerosis formation proximally to MBs, at least a different one than in arteries without bridged segments. In line with our results, observations from autopsy studies have suggested that although MBs can potentially promote atherosclerosis in the proximal arterial segments, this most probably is facilitated by other systematic factors.^27^ A recent study has demonstrated that bridged segments have lower pericoronary adipose tissue attenuation (i.e., with less inflammation) when compared with the non-bridged proximal segments, an effect that was evident only in patients with high atherosclerotic plaque burden.^28^

### Study Limitations

Our findings are limited by the fact that coronary flow was only simulated in the mid-end diastolic phase of the cardiac cycle and that it was considered to be laminar and not pulsatile. MBs have not been regarded to be of pathologic clinical significance since the majority of coronary blood flow occurs during diastole, while the systolic geometric distortion of MBs and exposure of the local endothelium to pathological flow patterns accounts for a much smaller part of the cardiac cycle. Simulation of blood flow in the systolic phase was not performed given that the anatomy acquisition and hence the geometry of the 3D reconstructed coronaries was in diastole. However, given the systolic compression, primarily of deep MBs, segmentation of the coronary lumen and further ESS estimation would be either not feasible or prone to errors. Use of invasive intravascular imaging (i.e., intravascular ultrasound; IVUS) could potentially allow for the assessment of the local hemodynamics in both phases of the cardiac cycle. Certainly, the power of the study was limited accounting for the small arteries sample, particularly with plaque presence. However, ESS was assessed in a moderate number of local regions of interest, thereby increasing statistical power. Our results can set the paradigm for a more comprehensive assessment of coronary atherosclerosis in patients with coronary anomalies by incorporating aside morphological evaluation, and the role of hemorheological and biomechanical forces.

## Conclusion

MBs are characterized by ESS homogeneity and normal ESS values, potentially explaining the lack of atherosclerosis within the bridged segment. Local ESS around the entrance of MBs did not fluctuate. Larger studies are warranted to assess whether ESS might not hold the protagonistic role in the pathophysiology of atherogenesis proximally to the MBs as suggested in the present study.
